# An analysis of doctoral prestige and faculty composition in Canadian mechanical engineering programs

**DOI:** 10.1371/journal.pone.0344491

**Published:** 2026-03-30

**Authors:** W. Brent Lievers, Katie A. Goggins, Marc Arsenault

**Affiliations:** 1 School of Engineering and Computer Science, Laurentian University, Sudbury, Ontario, Canada; 2 Center for Research in Occupational Safety and Health (CROSH), Laurentian University, Sudbury, Ontario, Canada; 3 School of Natural Sciences, Laurentian University, Sudbury, Ontario, Canada; Bar-Ilan University, ISRAEL

## Abstract

Faculty positions at institutions of higher education are limited resources for which there is a high level of competition. Previous studies have shown that a hierarchy of prestige exists within academia which leads to 80% of those positions being filled by the doctoral graduates from only 20% of institutions. The goal of the current study is to determine whether such a hierarchy also exists within Canadian mechanical engineering programs and to consider other demographic differences within their faculty complements. Information on over 1100 faculty members from the 38 accredited Canadian mechanical engineering programs were gathered from public sources, including their doctoral training institution, their year of graduation, and the name(s) of their faculty advisor(s). It was found that 22% of institutions were responsible for the doctoral training of 78% of faculty members. A PageRank analysis, coupled with k-means clustering, identified four tiers of doctoral prestige and demonstrated that few trainees are hired by a more prestigious institution. For example, only 9% of those trained at a Tier III institution were hired by a Tier II and none by a Tier I institution. Academic advisors also played an important role; 11 unique advisors had five or more of their trainees currently employed in those 38 programs. Only 16.7% of faculty members were female-presenting, lower than the 17.5% proportion of so-called “self-hires” (i.e., those employed by the institution where they did their doctoral training). This study provides analytical tools for quantifying institutional prestige as well as a demographic snapshot of Canadian mechanical engineering faculty that can be used to analyze other fields and jurisdictions. Further research in faculty hiring will benefit students looking to pursue careers in academia, as well as guide those responsible for ensuring equitable hiring practices.

## 1 Introduction

Faculty positions are a finite resource; there are a limited number of total positions in any given field. As public funding for institutions and programs decreases, and as the use of non-tenure track faculty increases, these numbers may be expected to shrink. Furthermore, job openings occur sporadically because only a small subset of the positions need to be filled at any given time. The elimination of mandatory retirement in many jurisdictions means that some members continue working into their 70s and beyond [1]. This behaviour is not a problem in and of itself, particularly given the training and time it can take to secure a position; however, it might slow the overall rate of faculty turnover and renewal. The scarcity of the positions, and the length of time that each position might be filled, means that hiring must be performed very carefully, purposefully, and equitably.

How one views the academic job market under these conditions will depend on the side of the hiring process on which one finds oneself. From the perspective of those hiring, there is a large pool of possible candidates with a greater supply than there is demand [2]. In 2015, only 15% of U.S. doctoral graduates obtained a faculty position within three years of obtaining their PhD [3]. At the same time, there is also increasing awareness that the hiring for these positions needs to be done in a fair and equitable fashion. Intrinsic factors such as race and gender have been shown to negatively affect one’s chances of being hired [4] while, at the same time, a so-called “leaky pipeline” reduces the number of potential candidates earlier in their training [5,6]. These barriers are seen as a negative on the basis of fairness, but also from the perspective that a diversity of ideas, backgrounds, and approaches improves the overall quality of the research and teaching within institutions of higher education. It is the same reason that hiring one’s own graduates — a practice sometimes pejoratively termed “academic inbreeding” — is often discouraged [7,8]. As a result, greater efforts are made when hiring to attract and increase the proportion of under-represented groups such as female faculty within engineering.

From the vantage point of those applying for positions, candidates recognize that there are performance- or merit-based factors that will determine one’s success. For example, research and teaching experience, publication records, and funding history are all considered by hiring committees. But there are also less obvious factors that affect one’s success in securing employment. For example, tiers of doctoral prestige have been identified in faculty hiring across multiple disciplines; institutions hire the graduates of comparable or higher-prestige institutions [9–12]. Research indicates that these patterns follow a Pareto Law with roughly 80% of faculty being hired from 20% of institutions [12–14]. Moreover, there are social and networking factors that must be considered. The ability to navigate or fit into the culture of a field has been shown to be related to career success [15]. Although it has been less studied, one’s thesis advisor and their status within the field might also be expected to affect one’s career trajectory. Identifying these underlying factors may be helpful for those trying to secure one of the limited number of available faculty positions.

The primary goal of this research is to identify whether training-based factors, such as the institution from which one obtains a doctorate degree or the advisor who supervises their research, influence academic hiring in Canada. To this end, faculty members associated with each of the 38 accredited mechanical engineering programs across Canada were identified from information available on the respective program websites. Public sources were then used to identify their employer and academic rank. Information about their doctoral training was also collected including the name of the granting institution, the year in which the degree was awarded, and the name(s) of their advisor(s). In addition, the researchers identified female-presenting faculty members. A modified PageRank algorithm [16] was combined with k-means clustering to quantify the tiers of doctoral prestige. Furthermore, the proportion of female-presenting faculty and self-hires within each faculty were compared. Finally, the *academic ages* of the faculty members, calculated as the number of years from the time they obtained their PhD to the date of data collection, were analyzed. These results will provide program hiring committees and academic units with a baseline against which to evaluate their performance in terms of equitable hiring practices. It will also help prospective faculty members understand the nuances of the highly competitive academic job market.

## 2 Methods

### 2.1 Data collection

Note that the terms *program*, *unit*, and *institution* will be used repeatedly throughout this article; each is intended to convey an increasingly broader scope. *Program* will refer to a degree offering such as mechanical engineering, *unit* will be limited to a School or Department (which may house multiple programs), and *institution* will include all units and programs within an entire university or college.

There are 40 accredited mechanical engineering programs in Canada listed under either *mechanical* (29), *mechanical systems* (1), or *génie mécanique* (10) on the Engineers Canada website [17]. L’Université du Québec à Trois-Rivières has three programs, split across two campuses, listed in those categories; these three programs were treated as one, resulting in a total of 38 institutions. Stand-alone mechatronics engineering programs, although related, were excluded. Simon Fraser University was not included for this reason as it has only a mechatronics engineering program but not a mechanical one.

Information about the faculty members was initially gathered from the unit websites of the various institutions. All snapshots of the faculty lists were taken during the week of August 4–10, 2024 for consistency. Every full-time faculty member was included in the analysis, including those in traditional tenure-track as well as teaching- or research-focused positions. Sessional, adjunct, cross-appointed, and emeritus faculty were excluded from the study.

Where units had multiple programs listed in their names, such as the *Department of Mechanical and Industrial Engineering* or *Department of Mechanical and Materials Engineering*, no effort was made to partition faculty; all were considered as belonging to “mechanical engineering” for the purposes of this study. Exceptions were made with institutions such as the University of Guelph, which have a single school of engineering, and faculty are listed as being associated with a particular program within that school. The list of the 38 institutions, the unit names, and the total number of faculty members identified in each are given in Table 1.

**Table 1:** Names of the institutions and units that house the 38 mechanical engineering programs studied. The numbers of relevant faculty members identified in each unit (*N*) are also listed.

Institution	Unit	*N*
British Columbia Institute of Technology	School of Energy	16
Carleton University	Department of Mechanical and Aerospace Engineering	51
Concordia University	Department of Mechanical, Industrial and Aerospace Engineering	60
Conestoga College	School of Engineering and Technology	12
Dalhousie University	Department of Mechanical Engineering	20
École de technologie supérieure	Département de génie mécanique	56
Lakehead University	Department of Mechanical Engineering	10
Laurentian University	School of Engineering and Computer Science	8
McGill University	Department of Mechanical Engineering	29
McMaster University	Department of Mechanical Engineering	30
Memorial University of Newfoundland	Department of Mechanical and Mechatronics Engineering	16
Ontario Tech University	Department of Mechanical and Manufacturing Engineering	20
Polytechnique Montréal	Département de génie mécanique	53
Queen’s University	Department of Mechanical and Materials Engineering	33
Royal Military College of Canada	Department of Mechanical and Aerospace Engineering	23
Toronto Metropolitan University	Department of Mechanical, Industrial, and Mechatronics Engineering	38
Université Laval	Département de génie mécanique	25
Université de Moncton	Département de génie mécanique	7
Université de Sherbrooke	Département de génie mécanique	32
Université du Québec en Abitibi-Témiscamingue	École de génie	11
Université du Québec à Chicoutimi	Département des sciences appliquées	14
Université du Québec à Rimouski	Département de mathématiques, informatique et génie	15
Université du Québec à Trois-Rivières	Département de génie mécanique	18
University of Alberta	Department of Mechanical Engineering	61
University of British Columbia	Department of Mechanical Engineering	50
University of British Columbia – Okanagan	School of Engineering	26
University of Calgary	Department of Mechanical and Manufacturing Engineering	45
University of Guelph	School of Engineering	17
University of Manitoba	Department of Mechanical Engineering	27
University of New Brunswick	Department of Mechanical Engineering	14
University of Ottawa	Department of Mechanical Engineering	26
University of Saskatchewan	Department of Mechanical Engineering	20
University of Toronto	Department of Mechanical & Industrial Engineering	65
University of Victoria	Department of Mechanical Engineering	31
University of Waterloo	Department of Mechanical and Mechatronics Engineering	63
University of Windsor	Department of Mechanical, Automotive & Materials Engineering	38
Western University	Department of Mechanical and Materials Engineering	32
York University	School of Engineering	21
		1133

Once the faculty members were identified, publicly available resources were then used to gather information about their training. These public sources include university websites, personal or lab websites, institutional libraries and thesis repositories, the ProQuest dissertation and thesis database, WorldCat, and public LinkedIn profiles. The information collected included the faculty member’s name, employer, and their academic rank. Details of their doctoral training were also collected including the institution that awarded their PhD, the year they received their degree, the title of their thesis/dissertation, and the name(s) of their doctoral advisor(s).

A subset of faculty members were identified as female-presenting for the purposes of examining gender differences in mechanical engineering faculty compositions. This categorization was made by the authors for each faculty member based on their names, photos, and/or pronoun usage in the publicly available resources. This approach has been used by other researchers when having individuals self-identify is impossible or impractical [18]. Gender information is presented in aggregate in this paper to avoid the possibility of an individual being publicly misgendered.

Understanding the relationship between employing and training institutions requires accounting for any mergers, name changes, or rebranding that have occurred at various universities. For example, Ryerson University was renamed Toronto Metropolitan University (TMU) in April 2022. Furthermore, the University of Ontario Institute of Technology (UOIT) and University of Western Ontario have rebranded as Ontario Tech University and Western University, respectively. These names were treated as synonyms. The Technical University of Nova Scotia (TUNS) merged into Dalhousie University in 1997, so graduates of the former were grouped with the latter as part of the analysis.

This data collection protocol was reviewed by the Laurentian University Research Ethics Board (LUREB). The study was deemed to be exempt from ethical oversight as it relies on publicly available information for which there is no reasonable expectation of privacy (REB file number 2024-046-01). Subsequently, consent was not sought from the individuals included in the analysis. Only the aggregated data underlying the graphs and figures presented in this paper are available for reuse as open data from the Borealis repository (consult the Supplementary Materials section for more details).

### 2.2 Analyses

#### 2.2.1 Doctoral training institution.

Various analyses were performed to understand the relationships between the institution where faculty obtained their doctoral training and their current employer. Previous work has found university hiring follows the Pareto principle, or 80/20 rule, where 80% of faculty were hired from 20% of institutions [13]. The proportion *x* of institutions, where 1−x of faculty were trained at those institutions, was calculated from the assembled data.

In addition to how many graduates are hired, one must also consider *where* those graduates are hired. Previous research has demonstrated that there is a hierarchy of prestige that exists in faculty hiring, with most graduates finding employment at equivalent or lower-prestige institutions [12]. Network analyses are a common way to identify the nature of those prestige rankings [9,10,19,20].

For this study, a modified form of the Google PageRank algorithm [16] was used to identify trends of prestige within faculty hiring patterns. The original PageRank algorithm calculates the importance of a webpage based on the number of inbound links to that page and the quality of the sources from which those links originate. Briefly, the page rank (*PR*) for a particular page (*p*_*i*_) is calculated by:


$$PR(p_{i})=\frac{1-d}{N}+d\cdot \sum_{p_{j}\in to(p_{i})}\frac{PR(p_{j})}{from(p_{j})}$$ (1)

where *d* is a damping factor, *N* is the total number of pages, *to*(*p*_*i*_) is the set of pages that link to page *p*_*i*_, *p*_*j*_ is one such linking page from the set of *to*(*p*_*i*_), and *from*(*p*_*j*_) is the total number of outbound links from page *p*_*j*_. Each page contributes an equal share of its own PageRank score to the other pages to which it links. An initial rank of 1/*N* is assigned to each page, and then the scores are updated iteratively using Eq. (1) until they stabilize to within a specified tolerance (or some maximum number of iterations is reached).

For the purposes of ranking the institutions, each faculty member was treated as a link *from* the employing *to* the training institution. Self-hires — those working at the same institution where they trained — were excluded, as were those trained at an institution outside of the 38 studied. The PageRank scores were then calculated using the algorithm implemented in the Python NetworkX package (version 3.1) and normalized to span the range [0,1]. The default damping factor of *d* = 0.85 was used. Links were weighted based on the number of individuals hired from a particular institution.

Rather than stratifying scores based on arbitrary thresholds [9,21] or external institutional rankings [11,22], a k-means clustering analysis [23] was performed to identify *K* tiers of prestige within the PageRank values. The value of *K* is normally required to be chosen or known in advance. To avoid the subjectivity of such a selection, it was assumed that the distribution of institutions would be roughly pyramidal, with each successively lower tier including more institutions than the one above it and fewer than the tier below it. Therefore, *K* was chosen based on the largest value that yielded the assumed pyramid shape (i.e., each lower tier has more members than the one above it).

Prestige analyses were performed on two sets of institutions. The first analysis included the 38 Canadian institutions that offer mechanical engineering programs. The second analysis focused only on the U15 Canada [24] institutions, which is an association of 15 research-intensive Canadian universities.

#### 2.2.2 Self-hiring.

The proportion of self-hiring for each institution was calculated as the percentage of faculty within each unit who had received their doctoral training at the institution where they were currently employed. Other connections such as undergraduate degrees, master’s degrees, or post-doctoral training might also be expected to contribute to where someone finds employment; however, those data were not collected. Employment history was also not considered when identifying self-hires. It is possible that someone began their career as a faculty member at another institution before returning to the location where they completed their doctoral training, for example, or vice versa. Only the employer at the time of data collection was considered.

#### 2.2.3 Doctoral advisors.

Because of the importance of the advisor–student relationship in graduate training, differences in hiring rates based on someone’s advisor is expected. To quantify this phenomenon, the number of former students, identified within the data set as current faculty members, was counted for each advisor. Fractional counting was used when a faculty member had multiple doctoral advisors (e.g., two advisors were each credited with 1/2 of the PhD). The sum of those values determined the total number of trainees each advisor had contributed to the faculty members captured in the data set; due to the fractional counting approach, an advisor’s total number of trainees may not be a whole number. It should be noted that not *all* an advisor’s trainees who have been hired into faculty positions will be considered in this analysis, only those included in the data set. Trainees with faculty positions at other institutions, or even different units within the 38 institutions considered, were not captured. A Pareto-style ratio (x:1−x) was calculated based on the total number of unique advisors in the data set.

#### 2.2.4 Gender and academic age.

Understanding the demographics of gender and age among mechanical engineering faculty members provides important insights into the profession as it existed in 2024. It also provides a useful snapshot to allow future evaluation of how that population changes with time.

The proportion of female faculty was calculated on an institutional and national basis. Although there are some limitations using the assumed gender, as opposed to allowing participants to self-identify, the former was deemed much more appropriate to ensure that the complete data set was available for the analyses.

Information on biological age was not collected, so “academic age” was calculated in order to evaluate the overall demographics of faculty members nationally. Previous studies have used various starting points to calculate this parameter including the year of a researcher’s first publication [25] or the year of their terminal degree [26]. In the current work, “academic age” was determined based on the difference between the data collection year (2024) and the year in which each individual was awarded their PhD. Faculty without doctorates, or those for whom data were missing, were excluded from the analysis.

Academic age was also analyzed based on academic rank. In this case, only those in traditional tenure-track positions were included, and only if a specific rank (assistant, associate, or full professor) was indicated. Gender differences in ages were also calculated to determine the proportion of female-presenting faculty within each group.

## 3 Results

The final data set included a total of 1133 faculty members from the 38 programs. The processed data used to create each of the graphs and tables presented in this section are available for reuse (see Supplementary Information).

### 3.1 Doctoral training institution

**Fig 1:**
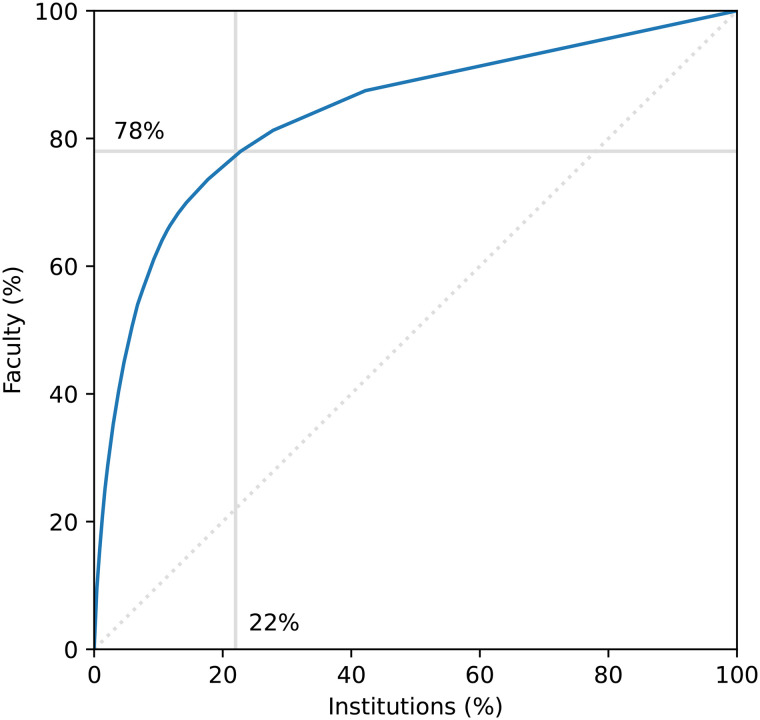
Cummulative distribution function indicating that a small minority of institutions (22%) contribute 78% of faculty, close to the 80/20 rule of the Pareto Principle.

The institutions where doctoral training was received were identified for 1096 of the 1133 faculty members. A total of 235 unique institutions were identified; however, an uneven distribution of hiring was observed based on where an individual received their training. Fig 1 indicates that only 22% of institutions were responsible for 78% of the mechanical engineering faculty. Table 2 lists the institutions that produced at least 4 faculty members.

**Table 2:** Number of faculty, *N*, trained per institution (minimum of 4).

Institution	*N*	Institution	*N*
University of Toronto	104	École Polytechnique Fédérale de Lausanne	8
University of Waterloo	64	University of California, Berkeley	7
Polytechnique Montréal	57	Simon Fraser University	7
McGill University	50	Memorial University of Newfoundland	7
Université Laval	40	Ontario Tech University	6
University of Alberta	36	Federal Institute of Technology (ETH) Zürich	6
University of British Columbia	34	Cornell University	6
Massachusetts Institute of Technology	29	Virginia Polytechnic Institute and State University	5
McMaster University	28	Université du Québec à Trois-Rivières	5
Queen’s University	25	University of California, San Diego	5
Concordia University	25	University of California, Davis	5
Western University	21	Toronto Metropolitan University	5
University of Victoria	21	University of Oxford	5
University of Manitoba	20	University of Illinois Urbana-Champaign	5
Université de Sherbrooke	19	Carnegie Mellon University	5
Dalhousie University	18	Yale University	4
University of Windsor	14	Université du Québec à Chicoutimi	4
University of Saskatchewan	13	Université de Montréal	4
University of Calgary	13	University of British Columbia – Okanagan	4
Stanford University	13	Delft University of Technology	4
University of Cambridge	13	University of Strathclyde	4
University of New Brunswick	12	Purdue University	4
École de technologie supérieure	11	Imperial College London	4
Carleton University	11	Harvard University	4
University of Ottawa	10	Georgia Institute of Technology	4
University of Michigan	9	École Centrale de Nantes	4
California Institute of Technology	9	Brown University	4

The identified faculty members received their training in 37 different countries. Almost 64% of Canadian mechanical engineering faculty were trained by institutions in Canada and the top five countries (Canada, United States, United Kingdom, France, Switzerland) account for 90% of faculty training (Table 3). It should be noted that training is distinct from the nationality of the individuals; the latter data were not collected.

**Table 3:** Number of faculty, *N*, trained per country (minimum of 4).

Country	*N*
Canada	699
United States	176
France	49
United Kingdom	47
Switzerland	14
Germany	13
China	9
Hong Kong	7
Singapore	6
Japan	6
Romania	5
Netherlands	5
Italy	5
Iran	5
India	5
Australia	5
Sweden	4
Russia	4

**Fig 2:**
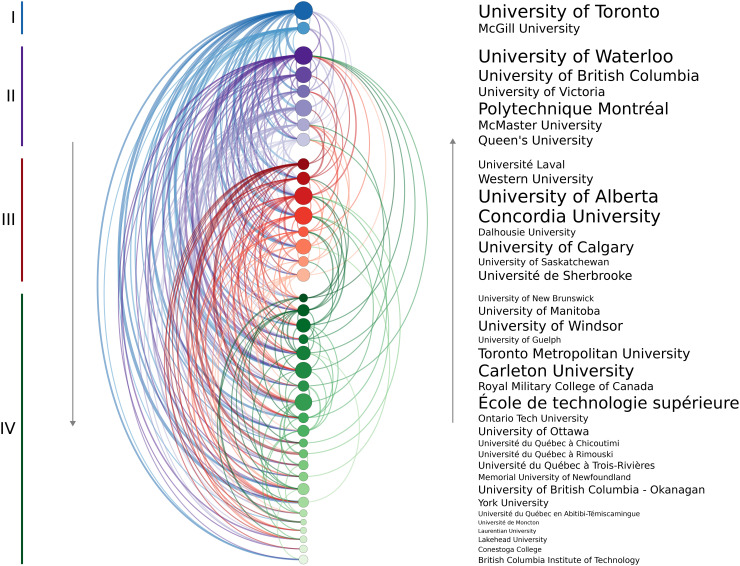
Arc diagram indicating the hiring relationships of mechanical engineering faculty within the 38 institutions. Each coloured arc represents a link from a training to an employing institution, with greater line weights indicating a larger number of faculty. Node size corresponds to the total number of faculty within the identified unit of that institution. The institutions are ordered by descending PageRank, with the colour groups corresponding to the four tiers of prestige uncovered using the *k*-means clustering with the assumed pyramid shape. Arcs on the left indicate hiring by a lower ranked institution and those on the right indicate a candidate was hired by a higher ranked one. Self-hires and other institutions were not included in the calculations.

The modified PageRank analysis revealed four tiers of “prestige” among the 38 institutions. The University of Toronto and McGill University ranked in the highest tier, with six institutions in Tier II, eight in Tier III, and the remaining institutions in Tier IV. A chord chart containing links from the training to the hiring institution is given in Fig 2, where arcs on the left indicate a move to a lower institution and those on the right indicate hiring by a higher prestige institution.

Table 4 summarizes the combined movement from one prestige tier to another. It should be noted that this table includes the effects of self-hiring whereas Fig 2 did not. For example, 18.2% of faculty members trained at a Tier I institution are currently employed by an institution in the same tier, while 18.2%, 22.7%, and 40.9% are employed by Tier II, III, or IV institutions. Conversely, only 3.9% of those trained at Tier II institutions moved up to Tier I despite the fact that there are more Tier II trainees in the data set (229) compared to Tier I (154). Not one individual trained at a Tier III or Tier IV institution moved up to a Tier I institution; instead, over half of faculty at Tier I were hired from institutions outside the group studied herein. These results again emphasize the challenge for trainees to be hired by higher prestige institutions.

**Table 4:** Number (percentage) of trainees that are hired by institutions of a given prestige tier, based on the prestige tier of their training institution. *Other* indicates institutions outside the 38 studied.

		Employing				
		I	II	III	IV	TOTAL
Training	I	28 (18.2%)	28 (18.2%)	35 (22.7%)	63 (40.9%)	154
	II	9 (3.9%)	84 (36.7%)	39 (17.0%)	97 (42.4%)	229
	III	0 (0.0%)	16 (9.0%)	83 (46.6%)	79 (44.4%)	178
	IV	0 (0.0%)	5 (4.6%)	14 (13.0%)	89 (82.4%)	108
	other	53 (12.4%)	117 (27.4%)	118 (27.6%)	139 (32.6%)	427
	TOTAL	90 (8.2%)	250 (22.8%)	289 (26.4%)	467 (42.6%)	1096

**Fig 3:**
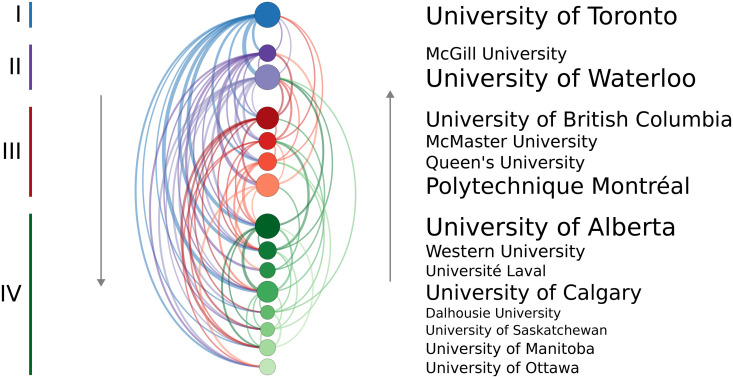
Chord diagram indicating the institutions from which faculty members at a given university obtained their PhD; however, only the U15 Canada group of research-intensive universities are included. Each coloured arc represents a link from a training to an employing institution, with institutions ordered by descending PageRank. The colour groups correspond to the four tiers of programs uncovered using the *k*-means clustering with the assumed pyramid shape. Self-hires and other institutions were not included in the calculations.

When only members of the U15 Canada group of research-intensive universities are included, four tiers are again identified (Fig 3); however, University of Toronto is now alone in the highest tier, with McGill University and the University of Waterloo in the second tier. Minor changes occur when comparing the complete and U15-exclusive rankings, with a few institutions dropping one tier. More notable is the performance of the University of Victoria given that it is the only high-prestige school (Tier II) in Fig 2 that is not part of the U15.

### 3.2 Self-hiring

**Fig 4:**
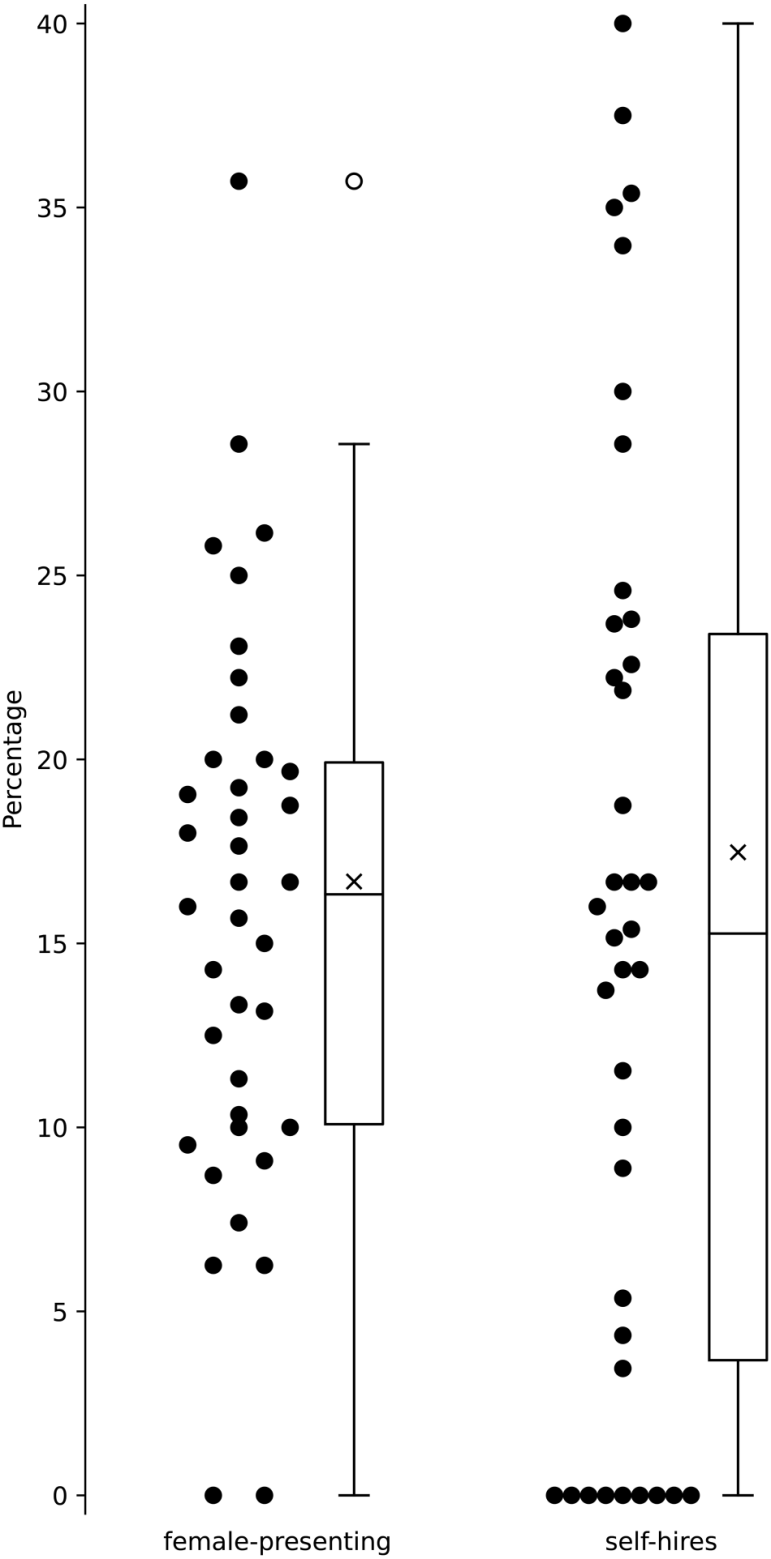
Institutional values and boxplots showing the proportion of female-presenting faculty and self-hires. The × indicates the national average. The open circles above the whisker plots indicate outliers outside the 5–95% percentile range.

The proportion of self-hiring was also found to vary dramatically by institution (Fig 4). Up to 40% of the faculty members in the units studied were currently employed at the institution where they had completed their doctoral degrees. The average for all institutions was 17.5%.

### 3.3 Doctoral advisors

**Fig 5:**
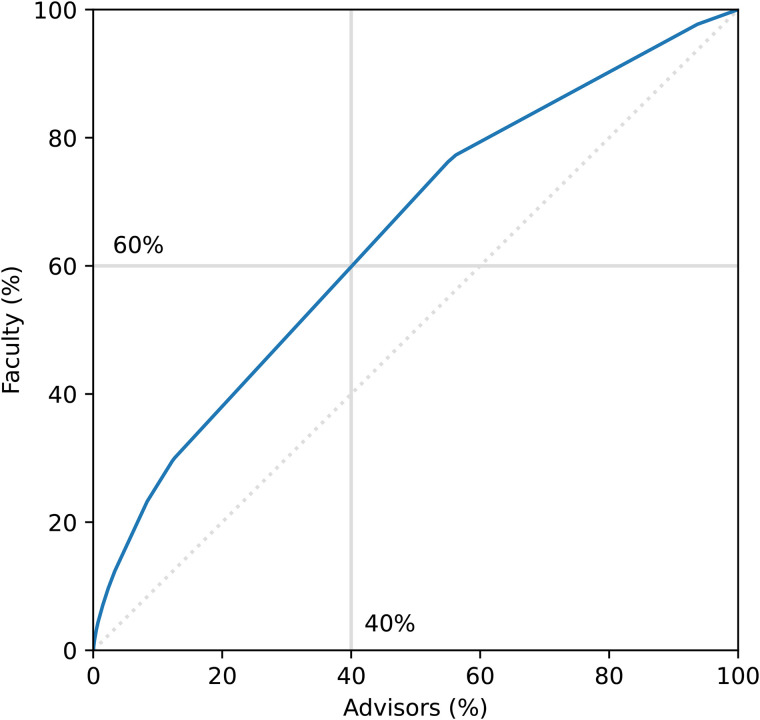
Cumulative distribution function indicating that faculty hiring is not evenly distributed across doctoral advisors, with 40% of advisors responsible for 60% of faculty. This value is a lower-bound estimate since only advisors with at least one hire are considered.

A total of 1076 unique doctoral advisors were identified for 1067 theses. A Pareto plot is shown in Fig 5 which uses fractional counting for theses with multiple advisors. The disparity is not as large as with institutions (Fig 1), as only 40% of advisors were responsible for 60% of faculty members. Some advisors trained as many as eight individuals currently employed as faculty members within the programs studied (7.5 using fractional counting). It must be understood, however, that the proportion of advisors only considers those who were identified in the data set. It does not include the other possible advisors who had doctoral students that were not subsequently hired as this would be much more difficult to quantify. As such, these values should be treated as a lower-bound estimate.

### 3.4 Gender and academic age

A total of 189 female-presenting faculty members were identified out of the 1133 included in the dataset, which represents a national average of 16.7%. The proportion of female-presenting faculty at each institution, however, ranged from 0 to 35.7% (Fig 4).

**Fig 6:**
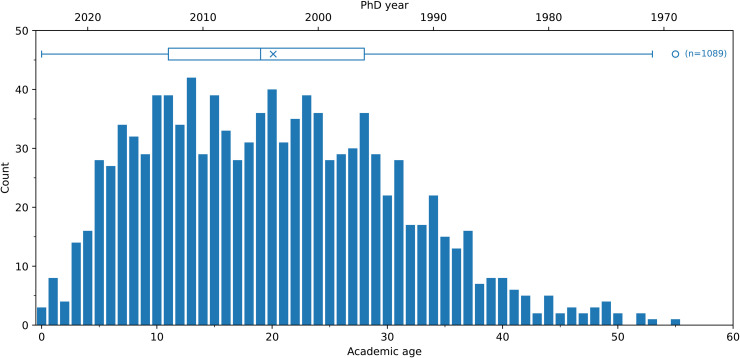
Histogram and box plot of the academic ages for all faculty based on years since they obtained their PhD relative to 2024. The × indicates the mean. Circles indicate outliers beyond the 95% percentiles shown by the box plot.

Information about the year in which doctoral degrees were awarded were available for 1089 faculty members. Academic age was calculated by subtracting the doctoral year from the reference year used for the data collection (2024). The age histogram (Fig 6) indicates a median of 19 years. The data is right-skewed, however, with 9.6% of active faculty members having obtained their PhDs in the 1980s or 1970s.

**Fig 7:**
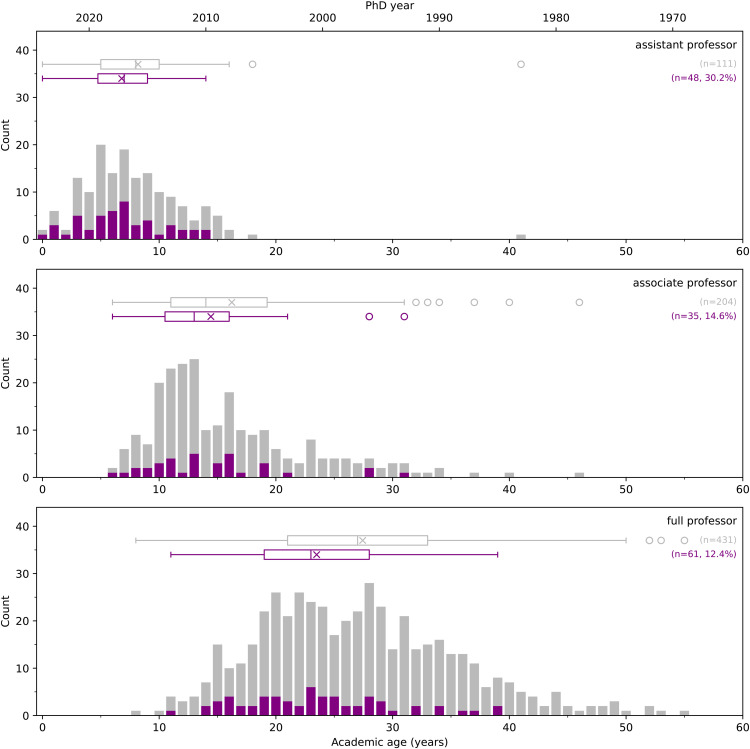
Stacked histograms and box plots for the academic ages for partitioned by rank (assistant, associate, full professor) and gender. The purple indicates the female-presenting faculty, with the percentage of the total included. The × indicates the mean. Circles indicate outliers beyond the 95% percentiles shown by the box plot.

When just tenure-track faculty are partitioned based on academic level, the expected shift in ages is seen as one moves from the rank of assistant, to associate, to full professor (Fig 7). Median ages of female-presenting faculty are less than their male counterparts at all levels, with the largest difference (≈4 years) at the full professor level. The proportion of female-presenting faculty decreases with increasing rank. Thirty percent of assistant professors in Canadian mechanical engineering programs are female-presenting, but only 12.5% at the rank of full professor.

## 4 Discussion

### 4.1 Prestige

Previous work has shown that faculty hiring is hierarchical; institutions tend to hire those who received their doctoral training from an institution that is more prestigious. This phenomenon has been documented in a wide variety of fields such as: sociology [9]; engineering [11]; political science [14]; and business, computer science, and history [12]. Although most of these studies have focused on hierarchies within the U.S. education system, similar trends have also been observed in other countries [10,21,22].

The current study confirms that this phenomenon also exists within Canadian engineering programs. Fig 2 illustrates that the vast majority of doctoral graduates move to a lower-prestige institution than the one where they received their doctoral training. Analyzing these training-hiring relationships, by combining the PageRank algorithm with k-means clustering, identified four tiers of doctoral prestige within Canadian mechanical engineering programs. By considering all the institutions within a particular tier, the current work also supports the idea that, in the rare cases someone is hired by a more prestigious university, it tends to only be by an institution within the same tier or the one above. Although this behaviour should be expected to emerge from the hiring-based definition of prestige used in this study, the magnitude to which preferential hiring occurs is notable. For example, only 9% of those trained at a Tier III institution work at a Tier II institution and none were hired by the top schools (Table 4). When analyzing only the U15 Canada group of research intensive institutions (Fig 3), four tiers were again identified and similar trends of limited upward mobility are observed.

To avoid the subjectivity associated with pre-selecting the number of clusters for the k-means analysis used to determine the tiers, a pyramidal shape was assumed and the value of *k* was chosen such that it was the largest value whereby each cluster had a greater number than the one above it. The fact that four tiers were again identified within the U15 Canada institutions suggests that this assumption may be obscuring subtle differences in the clusters when analyzing all 38 institutions. Alternative methods of objectively selecting the appropriate number of clusters should be considered in the future.

Almost two-thirds of faculty associated with Canadian mechanical engineering programs were trained at Canadian institutions, and 90% were trained in just five countries. Because the analysis was limited to the 38 Canadian mechanical engineering programs, it is impossible to determine the prestige of the non-Canadian institutions. The PageRank calculations used in Eq. (1) require knowledge of both faculty *hired by* and *trained by* that institution. Expanding the analysis to include non-Canadian programs would necessitate a substantial increase in the scope of the data collection. As a result, their influence on the prestige rankings has been ignored.

The results of both the full and U15-only analyses suggest that the modified PageRank algorithm introduced herein, which incorporates k-means clustering, is an effective tool for identifying patterns of institutional prestige. Other approaches include external evaluations such as the World University Rankings, which are then partitioned to determine prestige tiers [22]. Although PageRank has been used by other researchers [14], the authors believe that combining this approach with objective k-means clustering is a novel contribution to the analysis of institutional prestige.

Nevertheless, one of the main limitations of this analysis is that it does not account for the effects of program size or age. A program with more faculty members is more likely to have more PhD graduates. Assuming that the probability of being hired was the same for the doctoral graduates of all institutions, those programs with more graduates would appear to be more prestigious. This effect cannot be accounted for in the current analysis. Nevertheless, the fact that the prestige rankings are not simply based on decreasing number of faculty members (Fig 2) suggests that qualities beyond size alone are being measured.

It could likewise be argued that older, more established programs are also more likely to have a greater number of graduates in faculty positions because, over time, they have created a larger pool of graduates from which hiring could have been made. Because people are staying in these positions for many decades, older programs have a “first-mover” advantage over newer ones. A comparison of the employment outcomes of cohorts from multiple institutions is needed to account for these potentially confounding factors.

**Fig 8:**
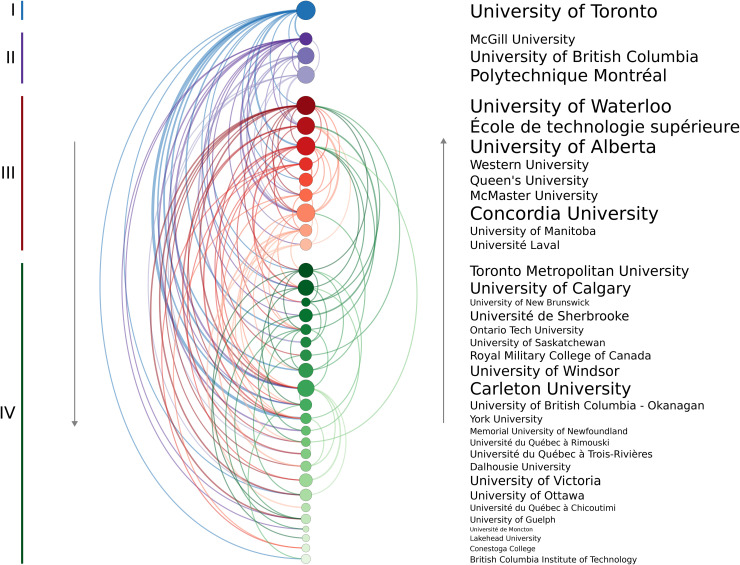
Chord diagram indicating the institutions from which faculty members at a given university obtained their PhD; however, only those who received their doctoral degrees from 2010–2019 are shown. Each coloured arc represents a link from a training to an employing institution, with institutions ordered by descending PageRank. The colour groups correspond to the four tiers of programs uncovered using the *k*-means clustering with the assumed pyramid shape. Self-hires and other institutions were not included in the calculations.

**Fig 9:**
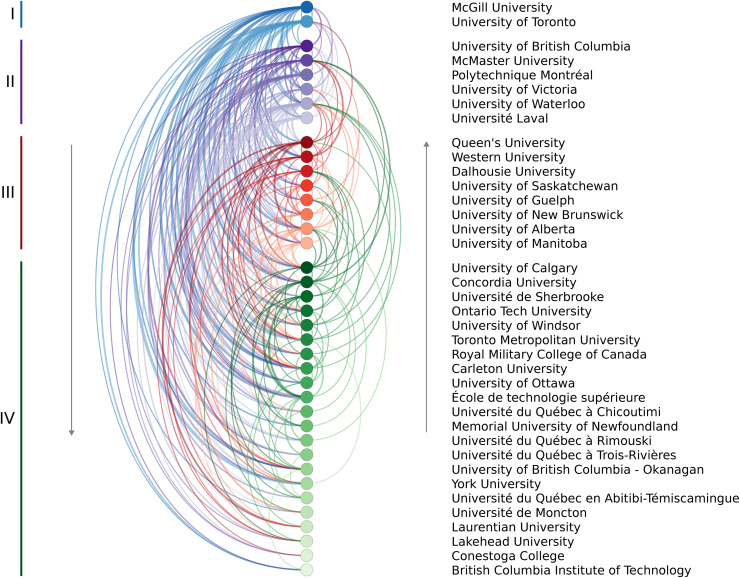
Chord diagram indicating the institutions from which faculty members at a given university obtained their PhD, where the weightings from an institution are normalized by the total number of faculty members of that institution. Each coloured arc represents a link from a training to an employing institution, with institutions ordered by descending PageRank. The colour groups correspond to the four tiers of programs uncovered using the *k*-means clustering with the assumed pyramid shape. Self-hires and other institutions were not included in the calculations.

The current data can be used to consider age and size effects, albeit in a limited way. For example, the effect of institutional age on prestige rankings can be investigated by including just a recent cohort of graduates. Fig 8 shows the prestige rankings calculated using only faculty members (*n* = 165) who received their doctoral degree during the decade 2010–2019. Although a few institutions have large increases (e.g., École de technologie supérieure) or decreases (e.g., University of Victoria) in prestige compared to the full analysis, the overall ordering is relatively stable between Figs 2 and 8. A similar analysis of the effect of size can be made by normalizing the number of links from one institution to another by the total number of faculty at the training institution given in Table 1. The changes in prestige rankings are again fairly subtle (Fig 9). These trends suggest that institutional age and size may not be as important a factor as other potential variables. Nevertheless, the future study proposed earlier, comparing the employment outcomes of doctoral cohorts from multiple institutions, would better account for size and age effects.

Another limitation of the prestige analysis is that it does not account for individuals’ employment history. Increased research mobility, the ability to move to different institutions for training or employment, has been shown to be associated with increased research productivity [27]. Therefore, mobility can reasonably be expected to contribute to employment success due to the importance that hiring committees place on research output. For example, some individuals may have started at lower-prestige institutions and relocated over time to higher-prestige ones. These moves will not be captured as the current data only provide a snapshot of what existed in 2024. Similarly, how geographic variables such as proximity to family, or constraints due to spousal/partner employment, factor into career decisions have not been considered in this study [28].

Finally, it should be emphasized that an institution’s doctoral prestige is independent of the quality of the undergraduate education it provides. Some of the 38 institutions do not offer graduate programs, which means that they will always be in the lowest tier of prestige, despite offering high-quality undergraduate training. All accredited mechanical engineering programs were included in the analysis, even those that do not provide doctoral training, because the latter still hire doctoral graduates from other institutions. Prospective students should not use these rankings, which are based on doctoral training and hiring, to make decisions about where to pursue their undergraduate degrees; however, these rankings are of value for those considering doctoral training, particularly those who wish to pursue a career in academia.

### 4.2 Self-hiring

Hiring one’s own graduates is a relatively common practice. Previous research indicates that 4–50% of Canadian engineering faculty members received their doctoral training at the institution that now employs them [29]. The current analysis of mechanical engineering programs exclusively found a national average of 17.5%, and institutional values that varied between 0 and 40%.

Although self-hiring is often viewed as a negative practice, due to the assumption that it is associated with a greater homogeneity of thought and expertise, one should be cautious in drawing such conclusions. The current study only considers doctoral training and employment institutions. Individuals currently working at the institution where they obtained their PhD may have trained in another program or unit, may have completed an undergraduate and/or a Master’s degree elsewhere, may have performed post-doctoral training elsewhere, and may have been faculty members at another institution before returning.

### 4.3 Doctoral advisors

Relatively little attention has been paid in the literature to the role that doctoral advisors play in subsequent employment within academia compared to the importance of the institution where they received their doctoral training. Survey- and interview-based studies indicate that more active mentorship by advisors is associated with greater job success generally and with a greater likelihood of securing tenure-track positions specifically [30–32]. Information tracking which advisors have students who successfully secure faculty positions is lacking. Part of the reason for this omission is the challenge of identifying someone’s advisor(s) as compared to simply identifying the institution where they received their doctorate. One of the novel aspects of this work is that this information was collected.

Doctoral advisors were identified for 1067 Canadian mechanical engineering faculty members. It was found that 40% of the 1076 unique advisors accounted for almost 60% of those hired. This result suggests that the identity of one’s advisor is an important factor in employment success, even if the effect is not as strong as that of the training institution. However, it must be understood that both approaches consider only the unique values within the data set and do not account for the total number of possible advisors or institutions. Although both will be underestimations of the true values, the advisor results can be expected to be more sensitive to this effect due to the fact that each possible doctoral institution pools the graduates of multiple possible doctoral advisors. Caution is required when interpreting these data. For example, 11 individuals had at least five or more of their former trainees currently employed in faculty positions captured by the data set. The total number of PhDs trained by these advisors was not considered, nor was the proportion of their trainees that were hired into faculty positions. As with the institutions themselves, there are likely size effects that need to be considered in future analyses.

### 4.4 Female faculty

The proportion of female-presenting faculty within Canadian mechanical engineering programs ranged from 0 to 35.7%, with a mean value of 16.7%. In 2022, Engineers Canada reported that 19.5% of engineering faculty, across all programs, were women [33]. As a point of comparison, Engineers Canada indicates that 23.3% of undergraduate engineering degrees were awarded to female-identified students in 2022 [33]. They report a similar number (25.8%) for doctoral degrees. Of course, the proportion of female students is not constant across disciplines. Only 15.8% of undergraduate mechanical engineering degrees were awarded to women, which makes the faculty numbers reported in the current study consistent with undergraduate enrollment levels.

Fig 7 indicates that the proportion of women was highest for assistant professor positions (30.2%), and decreased at the ranks of associate (14.7%) and full professor (12.5%). These numbers are similar to the 2022 statistics published by Engineers Canada for all programs [33]: 38.5% (assistant), 18.7% (associate), and 13.5% (full professor). While these numbers bode well for the future, the issue of retention will be important to whether the demographic inertia associated with the existing complement can be overcome to achieve greater representation [34].

It should be noted that the faculty members included in the survey did not have the opportunity to self-identify. Instead, they were classified as female-presenting by the researchers based on cues such as their name, photo, or pronoun usage in the public resources consulted. This is an approach that has been used in other studies where having people respond individually is not practical [18]. Although this methodology does not allow for the spectrum of gender to be explored, it does provide some insight into the proportion of women in engineering faculty positions. Interestingly, the proportion of female faculty is lower on average than the proportion of self-hires; institutions are more likely to hire an alumnus than they are to hire a woman.

### 4.5 Academic age

The academic age of faculty members was calculated by subtracting the year in which they obtained their PhD from the data snapshot year (2024). Although people may obtain their degrees at different chronological ages, “academic age” provides a useful estimate of faculty demographics.

Canadian mechanical engineering faculty members have a median academic age of 19 years and a mean of 20 years; however, the distribution exhibits a long-tail. Almost 25% of current faculty are still active 30 or more years after completing their PhD. This trend is important to consider from the perspective of faculty planning and renewal. It also underscores the importance of purposeful, effective recruitment and hiring, since candidates may occupy those positions for three or more decades.

### 4.6 Future work

The current work has considered multiple variables that might affect someone’s hiring prospects, including their doctoral institution, doctoral advisor(s), and gender; however, these factors have only been considered in isolation. A more rigorous, multi-variate analysis would be beneficial to apply an intersectional lens [35,36] to the issue of faculty hiring and understand the interactions between these and other potential variables such as race/ethnicity [37], mobility [27], family or relationships [28], and more. Even then, such an analysis would only consider those who had successfully secured a faculty position; it would ignore so-called “leaky pipeline” barriers that cause attrition earlier in the training process [5,6]. A more expansive data collection, along with a larger sample size, would be needed to detect the subtle roles and interactions of these effects on faculty hiring.

Institutional prestige is in many ways an academic form of career mobility. Sociologists have long generated mobility tables [38], similar to the one shown in Table 4, to examine outcomes such as children’s careers compared to those of their parents. Various statistical tools have been developed for the analysis of mobility tables generally [39], as well as some specific to faculty hiring [40]. Although not used in this current study, these techniques could be applied in future work to better quantify the probability of moving from a training to employing institutions with different levels of prestige.

Finally, thought must also be given to how these results can or should be incorporated into hiring practices. Little attention has been given in the literature on how address institutional bias specifically; however, many of the techniques used to eliminate other forms of hiring inequity might also be applicable. For example, training for hiring committees that covers implicit bias has been shown to improve the equity of the hiring process [41]. The way that jobs are advertized and promoted can also improve the diversity of applicants. The wording of the job posting itself can implicitly discourage some populations from applying [42]. Similarly, active recruiting strategies, including simply ensuring the job posting is published where underrepresented groups might see it, has been shown to increase the pool of applicants [43]. Blinding committee to certain pieces of information has also proved beneficial [44], but may be difficulty in faculty hiring.

### 4.7 Conclusion

Information on over 1100 faculty members from 38 Canadian mechanical engineering programs was collected and analyzed. It was found that 78% of faculty were trained by just 22% of institutions. This result contributes to a growing body of evidence, across multiple fields and jurisdictions, of the importance of prestige in faculty hiring. A novel stratification process was introduced which identifies tiers of prestige by combining PageRank network analysis with k-means clustering. Even when institutions within a tier were pooled, a strong preference for hiring from more prestigious institutions was observed. A similar trend was also demonstrated for doctoral advisors for the first time; however, the effect was more modest (60% of faculty trained by 40% of advisors). The demographic data reported provide an important snapshot of the composition of mechanical engineering faculty in Canada, in terms of its academic age, proportion of female-present faculty, and the rate at which self-hiring occurs. These results can serve as a valuable point of comparison when considering the faculty of other academic fields or nations. Taken together, the current work provides a set of tools and baseline data for analyzing factors which affect faculty hiring. Awareness of these biases can help improve the equity of hiring committee decisions and also provide valuable market information for students pursuing a career in academia.
